# Reduced coronary collateralization in type 2 diabetic patients with chronic total occlusion

**DOI:** 10.1186/s12933-018-0671-6

**Published:** 2018-02-08

**Authors:** Ying Shen, Feng Hua Ding, Yang Dai, Xiao Qun Wang, Rui Yan Zhang, Lin Lu, Wei Feng Shen

**Affiliations:** 1 Department of Cardiology, Rui Jin Hospital, Shanghai Jiao Tong University School of Medicine, Shanghai, 200025 People’s Republic of China; 2 Institute of Cardiovascular Disease, Shanghai Jiao Tong University School of Medicine, 197 Rui Jin Road II, Shanghai, 200025 People’s Republic of China

**Keywords:** Coronary artery disease, Type 2 diabetes mellitus, Coronary collateral circulation, Chronic total occlusion

## Abstract

**Background:**

The extent of coronary collateral formation is a primary determinant of the severity of myocardial damage and mortality after coronary artery occlusion. Type 2 diabetes mellitus (T2DM) represents an important risk factor for impaired collateral vessel growth. However, the mechanism of reduced coronary collateralization in type 2 diabetic patients remains unclear.

**Methods:**

With the reference to the recent researches, this review article describes the pathogenic effects of T2DM on collateral development and outlines possible clinical and biochemical markers associated with reduced coronary collateralization in type 2 diabetic patients with chronic total occlusion (CTO).

**Results:**

Diffuse coronary atherosclerosis in T2DM reduces pressure gradient between collateral donor artery and collateral recipient one, limiting collateral vessel growth and function. An interaction between advanced glycation end-products and their receptor activates several intracellular signaling pathways, enhances oxidative stress and aggravates inflammatory process. Diabetic condition decreases pro-angiogenic factors especially vascular endothelial growth factor and other collateral vessel growth related parameters. Numerous clinical and biochemical factors that could possibly attenuate the development of coronary collaterals have been reported. Increased serum levels of glycated albumin, cystatin C, and adipokine C1q tumor necrosis factor related protein 1 were associated with poor coronary collateralization in type 2 diabetic patients with stable coronary artery disease and CTO. Diastolic blood pressure and stenosis severity of the predominant collateral donor artery also play a role in coronary collateral formation.

**Conclusions:**

T2DM impairs collateral vessel growth through multiple mechanisms involving arteriogenesis and angiogenesis, and coronary collateral formation in patients with T2DM and CTO is influenced by various clinical, biochemical and angiographic factors. This information provides insights into the understanding of coronary pathophysiology and searching for potential new therapeutic targets in T2DM.

## Background

**Fig. 1:**
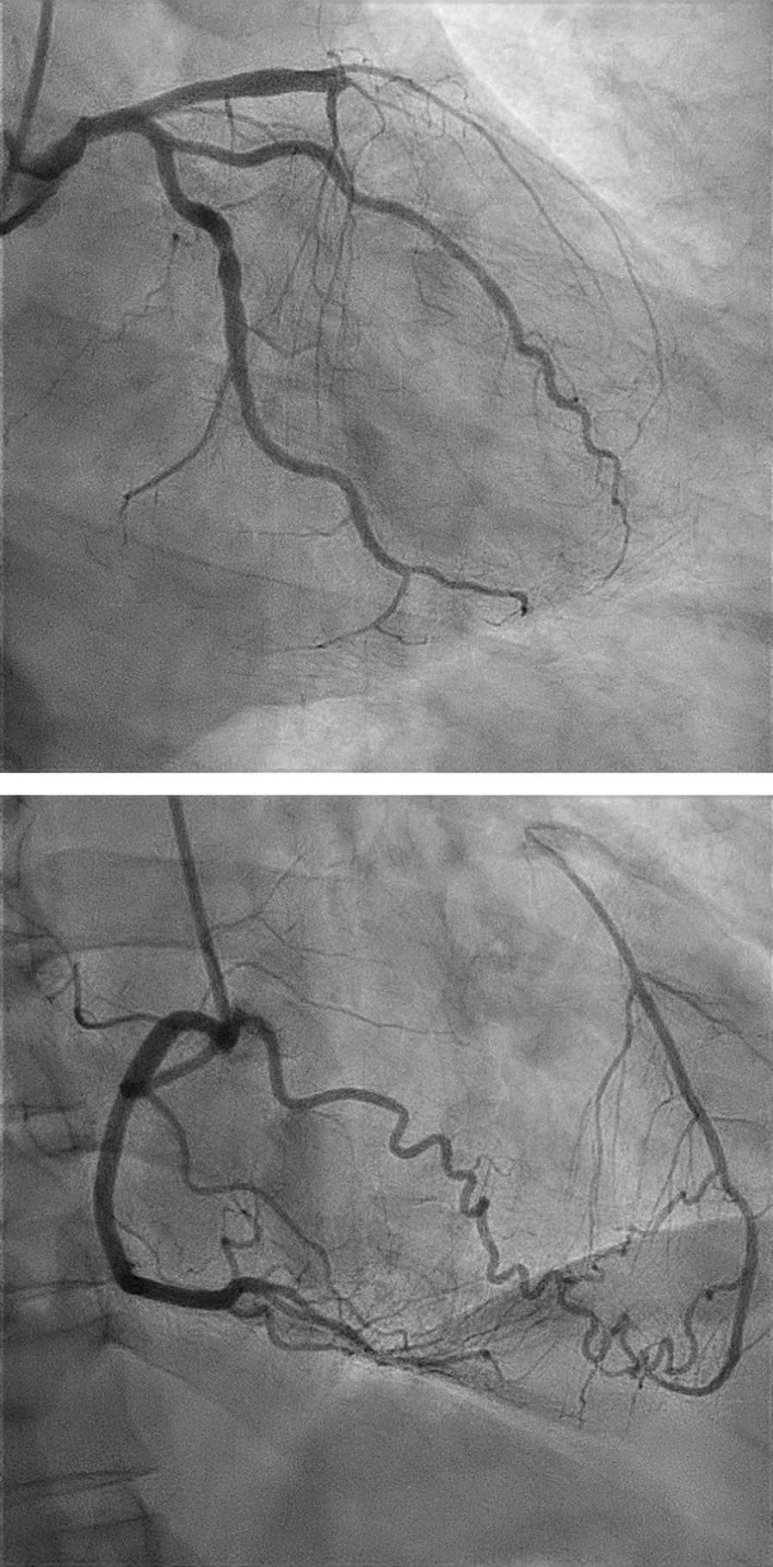
Coronary angiogram of a 58-year-old patient with stable angina. Upper: total occlusion of proximal left anterior descending artery; bottom: well-formed collaterals (Rentrop grade 3) supplied by the right coronary artery

In the human heart, vascular channels with lumen diameter ranging from 40 to 200 μm link large conductance coronary arteries to one another. These interconnecting arteriolar networks are called coronary collaterals [1, 2]. Abundant evidence indicates that when the proximal part of a major epicardial coronary artery is transiently or permanently occluded, the development of coronary collateral circulation serves as a natural conduit system bridging the occluded coronary vessels [3, 4]. Although these anastomoses are often incapable of restoring flow to normal levels, well-developed coronary collaterals could, at least partially, supplies the downstream perfusion area via the arteriolar connection (Fig. 1), thereby preventing or alleviating myocardial ischemia, reducing infarct size, protecting left ventricular function, and even decreasing mortality [5, 6].

Epidemiological data frequently demonstrate that type 2 diabetes mellitus (T2DM) is increasingly prevalent and represents an important risk factor for cardiovascular disease involving arteries and/or capillaries [7]. Compared with non-diabetic patients, those with T2DM often have more severe and diffuse coronary atherosclerosis, more complicated revascularization procedures (percutaneous coronary intervention [PCI] or coronary bypass grafting surgery), and less favorable long-term outcomes (e.g., higher rates of in-stent restenosis, stent thrombosis, and coronary atherosclerotic disease progression) [8]. In fact, cardiovascular disease remains the major cause of death for almost three quarters of type 2 diabetic patients, in which impaired coronary collateral formation may play a role [9].

In this review, we will describe the effects of T2DM on collateral vessel growth and discuss the role of clinical and biochemical factors as possible markers of reduced coronary collateralization and their clinical relevance in type 2 diabetic patients with stable coronary artery disease and chronic total occlusion (CTO), with the reference to the recent researches.

## Potential mechanisms of impaired collateral growth in diabetes

In adult organisms, the compensatory growth of blood vessels under ischemic conditions is an appreciated response, which can be achieved in two distinctive ways (i.e., arteriogenesis and angiogenesis) [10]. The process of arteriogenesis is stimulated by physical force and accompanied with enlargement and maturation of pre-existing arterioles (i.e., arterialization of the capillary bed). Briefly, when a coronary artery becomes completely occluded, the pressure gradient across the collaterals is elevated, which results in an increase in blood flow velocity and tangential fluid shear stress imposed on the endothelium, leading to a series of cellular response, including modulation of cell adhesion molecules that would in turn facilitate adhesion and transmigration of circulating mononuclear cells to sites of arterial formation. These cells then become activated and secrete matrix-degrading proteinases, leading to outward arterial remodeling. They also release other cytokines that contribute to the growth of arteriolar collaterals. Recent studies have shown that many genes related to inflammation, transcription, and neovascularization are significantly upregulated in the ischemic regions and associated with collateral growth [11]. The oxygen gradient over the collateral vessels is increased in patients with a less matured collateral circulation and related to local levels of pro-arteriogenic cytokines [12, 13]. Angiogenesis, sprouting of new capillaries from the pre-existing vessels, is induced by hypoxia-inducible factor 1-α and driven largely by vascular endothelial growth factor (VEGF) released either by ischemic tissues or by inflammatory cells. Angiogenesis is entirely regulated by a balance of pro- and anti-angiogenic factors [3]. Although formation and maturation of blood vessels are dependent on endothelial and vascular smooth muscle cells, and affected by various growth factors and inflammatory cytokines, the increase in diameter via arteriogenesis weights much more than the number of newly formed capillaries via angiogenesis [1–3].

The mechanism of reduced coronary collateralization in T2DM remains unclear and is likely multifactorial. Although the presence of a chronic coronary total occlusion would be expected to significantly decrease intracoronary pressure distal to the occluded segment which could promote arteriogenesis, there exists a trend towards severe coronary atherosclerosis in type 2 diabetic patients as manifested by long and diffuse lesions and small vessel disease, which could reduce pressure gradient between the collateral donor artery and collateral recipient one, and therefore, limits collateral vessel growth and function. The PROSPECT study with gray-scale and radiofrequency intravascular ultrasound showed that type 2 diabetic patients often have coronary atherosclerotic lesions characterized by a large necrotic core, thin-cap fibroatheroma, and high calcium content, especially for those with a longer duration of T2DM and poorer glycemic control [14]. These coronary lesion characteristics favor plaque instability and degradation, and predict future major adverse cardiac events independent of myocardial ischemia [15]. Previous studies have revealed that glucose fluctuations provoke oxidative stress that leads to endothelial cell dysfunction, progression of coronary atherosclerosis, and plaque vulnerability [16, 17]. These results suggest that there may be a symbiotic relationship between vulnerable plaque and T2DM. Recently, Hinkel et al. reported that diabetic human myocardial explants revealed capillary rarefaction and pericyte loss compared to nondiabetic explants. Moreover, they found that in a diabetic pig model, hyperglycemia induced microvascular rarefaction in the myocardium even without ischemia, and capillary density further decreased in chronic ischemia hearts [18]. These observations highlight that T2DM destabilized microvascular vessels of the heart and may impair the responsiveness of ischemic myocardium to proangiogenic factors. Data from prior studies have also shown a pronounced increase of collateral resistance, adverse functional and structural remodeling of the coronary arterioles, and obliteration of pre-existing blood vessels in T2DM [19–21]. All these changes jointly lead to reduced arteriogenic property in type 2 diabetic patients.

Chronic hyperglycemia and altered redox state in diabetes increase the formation and accumulation of advanced glycation endproducts (AGEs). Binding of AGEs to receptor for AGEs (RAGE) activates several intracellular signaling pathways including activation of mitogen activated protein kinase (MAPK), p21^ras^ and NF-κB translocation, resulting in enhanced oxidative stress and upregulation of many inflammatory genes [22]. Furthermore, overexpression of RAGE has negative effects on endothelial function, neointima formation, and angiogenesis [23]. Additionally, in a diabetic setting, pro-angiogenic factors including VEGF, fibroblast growth factor (FGF), and other collateral vessel growth related parameters are altered. There exists endothelial dysfunction characterized by decreased synthesis of nitric oxide, increased expression of endothelin-1 and adhesion molecules, elevated basal oxidative stress or more oxidative redox state, and increased vascular permeability [24]. Impaired monocyte/macrophage recruitment has been shown to be responsible for reduced collateral growth under diabetic conditions [25], and cytokines (e.g., transforming growth factor β, tumor necrosis factor [TNF]-α, monocyte chemotactic protein [MCP]-1, C-reactive protein [CRP], interleukin-8 and interleukin 20) and cell-extracellular matrix interaction may also play a role [24]. It has been shown that downstream VEGF receptors (VEGFRs) and Rho/Rho kinase pathway are important in the regulation of collateral development. Soluble VEGFR-1 is a negative endogenous modulator of angiogenesis by binding and sequestering VEGF. Increased expression and secretion of soluble VEGFR-1 prevents in vivo and in vitro capillary growth and angiogenesis [26]. The expression of soluble VEGFR-1 is decreased in hypoxia status and this protein molecule is degraded by local matrix metalloproteinase-7 to allow VEGF to escape sequestration in ischemic lesions [27]. In addition, soluble VEGFR-2 exhibits anti-lymphangiogenic property and its serum levels are related to insulin resistance in patients with metabolic syndrome, whereas the biological effect of soluble VEGFR-3 remains unclear [28]. These results suggest that VEGF-soluble VEGFR-1 mechanism is crucial to physiological homeostasis of vasculature and modulation of pro- and anti-angiogenesis. We have demonstrated elevated serum soluble VEGFR-1 and soluble VEGFR-2 levels and remarkably reduced VEGF and placenta growth factor levels in type 2 diabetic patients with CTO and low coronary collateralization [29], indicating a linkage of this negative regulator of angiogenesis to impaired coronary collateral formation. Previous studies have observed that myocardial expression of VEGFR-2 is reduced along with a down-regulation of its signal transduction in type 2 diabetic patients [30] and that AGEs inhibit VEGFR-1-mediated chemotaxis in diabetic monocytes [31]. Serum soluble VEGFR-1 level is increased in patients with T2DM [32], and diabetic condition aggravates vascular inflammation through amplifying RAGE-mediated mechanism [33]. In patients with T2DM, glycation of apoprotein A attenuates atheroprotective function of high-density lipoprotein (HDL), and, in contrast, glycation of apoprotein B reinforces low-density lipoprotein-induced inflammatory response [34, 35]. Thus, diabetic pathophysiology promotes an anti-angiogenic process and meanwhile mitigates pro-angiogenic factors in coronary vasculature during ischemia, jointly leading to impaired collateral growth.

Metabolic syndrome characterized by a cluster of risk components including abdominal obesity, insulin resistance, hyperglycemia, dyslipidemia and hypertension has been considered as one of the significant factors affecting adversely the development of coronary collateral vessels. In fact, this syndrome remained an independent risk factor for poor coronary collateralization even after adjusting for T2DM, and approximately 30–40% of these patients show little to no coronary collateral growth [36]. Previous studies indicated that an increasing number of component pathologies of the metabolic syndrome correlated with increasingly poorer coronary collateral development by angiographic grading systems [24]. Metabolic syndrome compromises vascular adaptations to ischemia, resulting in impaired coronary collateral growth. Central to this inadequate adjustment is impairment in endothelial function produced by oxidative stress, which also corrupts the signal transduction of growth factors [36].

## Factors influencing coronary collateralization in diabetes

Besides severity of coronary obstruction [37, 38], numerous factors that could possibly attenuate the development and biological function of coronary collaterals have been reported such as old age [39], traditional risk factors for coronary artery disease [40–44], hyperlipoprotein (a) [45], hyperuricemia [46] and elevated serum levels of CRP [47], TNF-a [48], N-terminal pro-brain natriuretic peptide [38] and mimecan [49], and high neutrophil/lymphocyte ratio [50]. In contrast, higher plasma levels of MCP-1 [51] or apelin [52] were associated with better coronary collateral development. The presence and extent of spontaneously visible coronary collaterals was also affected by plasma chemokine concentrations, as higher collateralization was associated with increased concentration of the angiogenic ligand and decreased concentrations of angiostatic ligands, and interferon-c [53]. It is known that diabetes mellitus aggressively induces atherosclerosis and may be more susceptible to myocardial infarction. Furthermore, the endogenous cardio-protective mechanism (such as collateral development) is blunted in type 2 diabetic patients, particularly for those with active smoking [54]. Recently, certain new factors have been studied in relation with reduced coronary collateralization in type 2 diabetic patients with CTO.

*Glycated albumin*(GA), a predominant early Amadori-type glycation protein in serum, serves as an alternative measure of dysglycemia over approximately 2–3 weeks to glycated hemoglobin (HbA1c), and is associated with the occurrence and severity of atherosclerosis in patients with T2DM [55]. It is also a prognostic marker for type 2 diabetic patients undergoing coronary artery stent implantation [56]. Shen et al. found that GA but not HbA1c levels in serum were significantly elevated in type 2 diabetic patients with low coronary collateralization than in those with high collateralization, and GA levels correlated inversely with semi-quantitative Rentrop collateral grade. Interestingly, after adjusting for age, gender, risk factors for coronary artery disease and renal dysfunction, a serum GA > 18.3% was more sensitive, compared with HbA1c > 7%, for predicting the presence of low coronary collateralization in type 2 diabetic patients with stable coronary artery disease and CTO [57]. In cellular experiment, GA promotes inflammation via activation of NF-κB pathway in endothelial cells, induces adhesion of monocytes to endothelial cells through enhanced transcription of the cell surface adhesion molecules, and stimulates vascular smooth muscle cell proliferation. GA could be also formed in a non-diabetic milieu triggered by inflammatory reaction. Previous studies have shown that GA induces endothelial dysfunction and produces pro-inflammatory effects in macrophages through augmentation of reactive oxide spices (ROS). Moreover, this glycated protein reflecting poor glycemic control impairs angiogenic function and elicits apoptosis of endothelial progenitor cells [58, 59]. These observations support a notion that GA may be either a risk factor or a marker for poor collateral growth in patients with T2DM.

*Cystatin C,* an endogenous anti-angiogenic factor, was considered as an emerging biomarker in cardiovascular disease and proved to be an important predictor for adverse outcomes among patients with coronary artery disease [60]. Recently, a large cohort study involving 866 patients with stable coronary artery disease and CTO demonstrated that low coronary collateralization corresponded significantly with elevated cystatin C levels in 498 patients with T2DM even after adjusting for multiple variables including renal function and levels of CRP. Patients with a cystatin C ≥ 0.97 mg/L had 2.37-fold increased risk of low coronary collateralization [61]. This suggests that anti-angiogenic cystatin C may be an indicator of coronary collateral formation in type 2 diabetic patients with stable coronary artery disease and CTO. It is evident that the effect of cystatin C levels on coronary collateralization reflects the net result of several pathophysiological processes. Cystatin C per se displays anti-angiogenic characteristics by reducing endothelial cell tubule formation and cysteinyl cathepsin activities [62]. Furthermore, cystatin C as a cysteine proteinase inhibitor is associated with cardiovascular risk factors as well as inflammation, which may promote vascular endothelial damage and cause low coronary collateralization [60].

These findings are also in line with the fact that cystatin C-based equation was superior to creatinine-based formula for estimating glomerular filtration rate (GFR) and identifying low coronary collateralization in type 2 diabetic patients with CTO [63]. Previous studies have frequently shown that renal dysfunction is strongly associated with poor coronary collateral growth and increased cardiovascular mortality, even when GFR is mildly decreased [64], suggesting that early detection of renal dysfunction is particularly important in the management of type 2 diabetic patients with coronary artery disease. Serum creatinine-based abbreviated Modification of Diet in Renal Disease (MDRD) formula is commonly used to estimate GFR, but it may lack accuracy to monitor kidney function in patients with early phase of renal impairment. Cystatin C, which is produced by all nucleated cells at a constant rate and has been considered to be a native anti-angiogenic factor, is freely filtered across the glomerular membrane and not influenced by age, sex, muscle mass, exercise or diet. Its serum level was used as an endogenous marker of renal function superior to serum creatinine [65]. Shen et al. found that in 302 patients with T2DM and CTO, GFR estimated using serum cystatin C (GFR_CYS_) correlated more closely with coronary collateral Rentrop score than that estimated using serum creatinine either by creatinine-based MDRD formula (GFR_MDRD_) or by Chronic Kidney Disease Epidemiology Collaboration equation (GFR_EPI_). The area under the curve of GFR_CYS_ was significantly larger compared with that of GFR_MRDR_ for predicting the presence of low coronary collateralization, along with a net reclassification improvement of 15.0%. After adjusting for possible confounding variables, a GFR < 90 mL/min/1.73 m^2^ estimated with cystatin C-based equation was more independently associated with low coronary collateralization. This suggests that cystatin C-based definition of renal dysfunction indicates a potential better clinical utility than creatinine-based formula for predicting poor coronary collaterals in type 2 diabetic patients with stable coronary artery disease [63]. Renal dysfunction which commonly occurs in patients with severe coronary artery disease and is partly reflected by elevated serum levels of cystatin C, adversely affects several components necessary for collateral growth through various regulatory mechanisms and gene expression [66].

*C1q tumor necrosis factor related protein (CTRP),* an adipokine family, has been shown to have diverse biological influences on cardiovascular system and disease process [67]. Among CTRP family members, adiponectin increases collateral formation and exhibits cardiovascular protective effects in patients with coronary artery occlusion [68]. CTRP3 exerts pro-angiogenic and cardioprotective effects in mouse with myocardial infarction [69]. In contrast, CTRP1, a CTRP family member with less structural similarity to adiponectin, is markedly upregulated by inflammatory cytokines in adipose tissue of Zucker diabetic rat and in adipocytes. The effects of CTRP1 and CTRP3 on coronary collateral growth have been assessed in T2DM patients with stable coronary artery disease and CTO [70]. Serum levels of CTRP1 and CRP were significantly higher in patients with low coronary collateralization than in those with high collateralization, but CTRP3 levels were similar in the two groups. Serum CTRP1 levels correlated with the number of diseased coronary artery and were independently associated with low coronary collateralization. Although elevated serum CTRP1 level is thought to be, in part, related to more profuse adipose tissue depot in type 2 diabetic patients with low coronary collateralization, further in vitro experiments which determine the effects of CTRP1 on angiogenic property of endothelial progenitor cells derived from culture of peripheral blood mononuclear cells showed that recombinant human CTRP1 protein decreased VEGFR-2 not VEGF and VEGFR-1 expression in a concentration -dependent manner in these cells. Thus, in vivo and in vitro data regarding the effect of increased CTRP1 level in serum and in cell culture medium may embody pro-inflammatory aspects of this protein [71].

*Diastolic blood pressure* determines the flow in the feeding coronary artery that supplies the collateral arteriolar network and generates the distal pressure within the occluded coronary segment because coronary perfusion occurs predominantly during diastole. A recent study has shown that diastolic blood pressure was related to Rentrop collateral grade in a U-shaped pattern in type 2 diabetic patients with CTO, and the optimal diastolic blood pressure ranges with the lowest risk of poor collateralization differ between type 2 diabetic patients (80–89 mmHg) and their non-diabetic counterparts (90–99 mmHg) [72]. This suggests that the influence of coronary vascular tone on collateralization may differ in the presence or absence of diabetes, and supports the concept that increased diastolic blood pressure may be, to a certain extent, a compensatory process to maintain normal coronary collateral flow, but too low or too high diastolic blood pressure could decrease the perfusion of coronary collaterals. Such a relationship between diastolic blood pressure and coronary collateralization is also consistent with the J-curve phenomenon relating the overly reduced or elevated diastolic blood pressure to adverse outcomes particularly in patients with T2DM [73].

*Stenosis of collateral donor artery* affects coronary collateral flow to the occluded bed. We calculated collateral flow index by simultaneous recording of central aortic pressure and intracoronary distal occluded pressure during PCI for CTO, and found that collateral flow index was strongly related to aortic diastolic and mean pressure even when a moderately stenotic lesion existed in the predominant collateral donor artery for patients with T2DM, as compared to a severe stenosis only for non-diabetic counterparts after adjusting for potential clinical and biochemical confounding factors [74]. The reason for different effects of donor artery stenosis severity on collateral flow relative to blood pressure between type 2 diabetic and non-diabetic patients remains unclear, but a likely explanation is the presence of more diffuse coronary atherosclerosis and microvascular disease in patients with T2DM [15–20].

## Clinical perspective

Since the AGEs-RAGE axis plays an important role in coronary collateral formation in patients with T2DM, it is a reasonable therapeutic target [19, 20]. Besides optimal glycemic control, the use of AGEs antagonists (such as aminoguanidine) has been shown to retard atherosclerotic process and promote collateral growth [22]. Pioglitazone exerts an angiogenic effect via Akt-VEGF pathway in a peroxisome proliferator-activated receptor-gamma (PPAR-γ) independent manner [75]. Several studies have highlighted the importance of an individualized blood pressure lowering strategy in type 2 diabetic patients with multivessel coronary disease [76], as any excessive decrease in systemic blood pressure with anti-hypertensive therapy before restoring anterograde flow of a chronic totally occluded lesion may compromise collateral recruitment and exacerbate myocardial ischemia especially for those with moderate or severe stenosis in the predominant collateral donor artery [77]. Similarly, vasodilator treatment could induce a redistribution of blood flow from the area supplied by an occluded artery towards the region supplied by the collateral donor artery (collateral steal) if the stenotic collateral donor artery cannot maintain the increased flow that occurs in two vascular beds [3, 78]. Although such a phenomenon is most likely to occur at the presence of a severe stenosis in the donor artery proximal to the origin of the collaterals, it might also occur at the presence of a moderate collateral donor artery stenosis in type 2 diabetic patients. Statin treatment was associated with high coronary collateralization assessed by the Rentrop collateral grading system due partly to reduced apoptosis and decreased release of sVEGFR-1 induced by proinflammatory cytokines and blood coagulation peptides [79]. The use of β blockers or If-channel inhibitor ivabradine reduces heart rate, improves fluid shear stress at the endothelial wall, and decrease catecholamine-mediated inflammatory response, favoring coronary collateral growth [80, 81]. However, angiotensin-converting enzyme inhibitor therapy may contribute to poor coronary collateral development in patients with CTO via inhibiting the expression of angiotensin II-induced growth factors such as VEGF, FGF, and platelet-derived growth factor [82].

Revascularization for a CTO appears to be associated with improved left ventricular function and clinical outcomes [83], and current evidence favors coronary artery bypass grafting as the preferred revascularization modality for type 2 diabetic patients with multivessel coronary disease, which is likely to reflect the more complete revascularization and global protection provided by arterial conduits against rapid atherosclerosis progression in PCI and untreated segment [84, 85]. Jane et al. found that in patients with CTO and well-formed collaterals, aggressive revascularization may reduce the risk of cardiac mortality and major adverse cardiac events [5]. Recently, Choi et al. reported that PCI may reduce the risk of cardiac mortality in non-diabetic patients but not in diabetic patients for the treatment of CTO [86]. However, Sanguineti et al. found that recanalization of a CTO was associated with improved long-term survival and suggests a greater reduction in cardiac death among diabetic patients [87]. We believe that the indication for recanalization of a chronic coronary total occlusion should be based not only on clinical manifestation of the patients but also on the morphology of occluded coronary lesions, the quality of collaterals, and myocardial viability. PCI aimed at improving collateral flow could do so by reducing proximal stenosis in the collateral donor artery, thereby increasing pressure at the collateral takeoff [88]. Further studies are needed to examine if determination of fractional flow reserve on moderate collateral donor artery stenosis could be useful for optimal therapeutic decision-making in type 2 diabetic patients with multi-vessel disease.

Previous clinical trials on restoration of perfusion through collateral growth using growth factors have failed largely because of an overlapping mechanism between arteriogenesis and atherosclerosis. In future, certain protective factors including endogenous soluble RAGE and vasostatin-2 which are decreased in patients with T2DM could be alternative therapeutic targets [89]. Emerging evidence suggests that microRNAs are implicated in a variety of physiological processes, including glucose homeostasis [90]. Circulating microRNAs in the plasma of patients with stable coronary artery disease and CTO may provide information about the coronary collateral capacity. For example, elevated miR-320 and miR-221 levels were indicative of endothelial dysfunction and accompanied with impaired angiogenesis in diabetes, whereas microRNA-126 was increased in patients with well-developed collateral circulation, along with VEGF levels [91]. In addition, although serum HDL was associated with the development of coronary collateral circulation [92], coronary atherosclerosis is more influenced by HDL quality than by its quantity in the diabetic condition. Glycation of apolipoprotein A-I and A-IV has been shown to be related to the presence and severity of coronary artery disease and plaque progression in T2DM [93–95]. Their effects on coronary collateral vessel growth in T2DM are currently investigated.

## Conclusions

T2DM adversely affects coronary collateral development through multiple cellular mechanisms on arteriogenesis and angiogenesis, and the formation of coronary collaterals in patients with T2DM and CTO is influenced by various clinical, biochemical and angiographic factors. Therefore, studies on the relationship between T2DM and coronary collateral circulation are clinically relevant in terms of understanding coronary pathophysiology in diabetes and searching for potential new therapeutic target in future.

## Abbreviations

AGEs: advanced glycation end products
CRP: C-reactive protein
CTRP: C1q tumor necrosis factor related protein
FGF: fibroblast growth factor
GA: glycated albumin
GFR: glomerular filtration rate
HbA1c: glycated hemoglobin
HDL: high-density lipoprotein
MAPK: mitogen activated protein kinase
MCP: monocyte chemotactic protein
MDRD: modification of Diet in Renal Disease
NF: nuclear factor
PCI: percutaneous coronary intervention
PPAR-γ: peroxisome proliferator-activated receptor-gamma
RAGE: receptor for advanced glycation end products
ROS: reactive oxide spices
T2DM: type 2 diabetes mellitus
VEGF: vascular endothelial growth factor
VEGFR: VEGF receptor
